#  Assays for Detection of Telomerase Activity 

**Published:** 2011

**Authors:** D.A. Skvortsov, M.E. Zvereva, O.V. Shpanchenko, O.A. Dontsova

**Affiliations:** Faculty of Chemistry, Lomonosov Moscow State University

**Keywords:** telomerase, telomerase activity assay, tumor diagnostics, physicochemical methods, polymerase activity assay, DNA determination

## Abstract

Progressive loss of the telomeric ends of chromosomes caused by the semi-conservative mechanism of DNA replication is an important timing mechanism which controls the number of cells doubling. Telomerase is an enzyme which elongates one chain of the telomeric DNA and compensates for its shortening during replication. Therefore, telomerase activity serves as a proliferation marker. Telomerase activity is not detected in most somatic cells, with the exception of embryonic tissues, stem cells, and reproductive organs. In most tumor cells (80–90%), telomerase is activated and plays the role of the main instrument that supports the telomere length, which can be used for the diagnostics of neoplastic transformation. This is the primary reason why assays regarding the development of telomerase activity have attracted the attention of researchers. Telomerase activity testing may be useful in the search for telomerase inhibitors, which have the potential to be anti-cancer drugs. Moreover, telomerase activation may play a positive role in tissue regeneration; e.g., after partial removal of the liver or cardiac infarction. All telomerase activity detection assays can be divided into two large groups: those based on direct detection of telomerase products, and those based on different systems of amplification of the signals from DNA that yield from telomerase. The methods discussed in this review are suitable for testing telomerase activity in different samples: in protozoa and mammalian cells, mixed cellular populations, and tissues.

##  WHAT IS TELOMERASE AND WHY DO WE NEED TO DETERMINE ITS ACTIVITY? 

In 1961, Hayflick and Moorhead demonstrated that a somatic cell culture has a limited life period (the Hayflick limit) [1]. In 1973, Olovnikov postulated that the number of cell divisions can be determined by the shortening of the telomeric ends of chromosomes [2], which play the role of a timing mechanism in cells. Telomeres protect the cell genome against degradation and participate in the meiotic pairing of chromosomes and the regulation of the transcription of the genes located in the pretelomeric region [3, 4]. Present in immortalized cells (with the ability of infinite division) is a mechanism that compensates for telomere shortening. In 1985, Blackburn and Greider discovered telomerase, an enzyme that elongates one of the telomere chains [5].

**Fig. 1:**
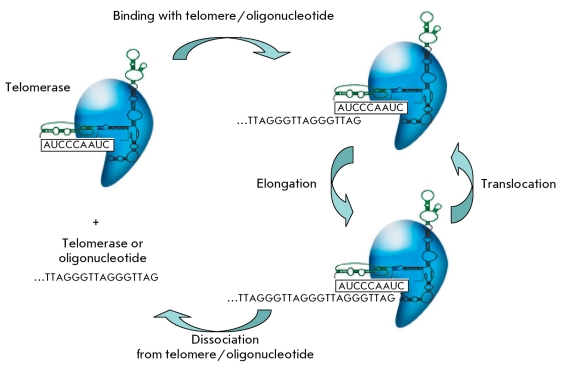
General functional scheme of the telomerase complex.

Telomerase is an RNA–protein complex; its main components are an RNA-matrix for telomere synthesis (TERC), which also has a structural function, and reverse transcriptase (TERT) [6]. The telomerase complex (telomerase) binds with a telomere or an oligonucleotide, the sequence of which may differ from the telomere sequence (telomere-imitating oligonucleotide), and synthesizes a short DNA fragment (in mammals, GTTAGG). Next, telomerase moves to the telomeric end (translocation) and again synthesizes a DNA fragment ( *Fig. 1* ). Telomerase activity is proportional to the total amount of DNA that it synthesizes, while the processivity is in proportion to the length of the fragments synthesized.

Telomerase activity is a marker of the proliferative activity of cells. Telomerase components may perform functions that are independent of the arrangement of the active complex. For example, hTERT (human TERT), regardless of the presence of hTERC (human TERC), may function as an RNA-dependent RNA-polimerase [7]. An enhancement of hTERC expression does not necessarily coincide with the emergence of telomerase activity [8]. hTERC inhibits protein kinase ATR, the targets of which are the known tumor growth suppressor p53 and CHK1 protein kinase at a checkpoint control, which is part of the system of signal transmission from the damaged DNA. A decrease in the hTERC level leads to stoppage of the cell cycle at G1 and G2 phases as a result of the activation of the p53 protein and CHK1 protein kinase. However, the mechanism of this activity has yet to be revealed [9].

**Fig. 2:**
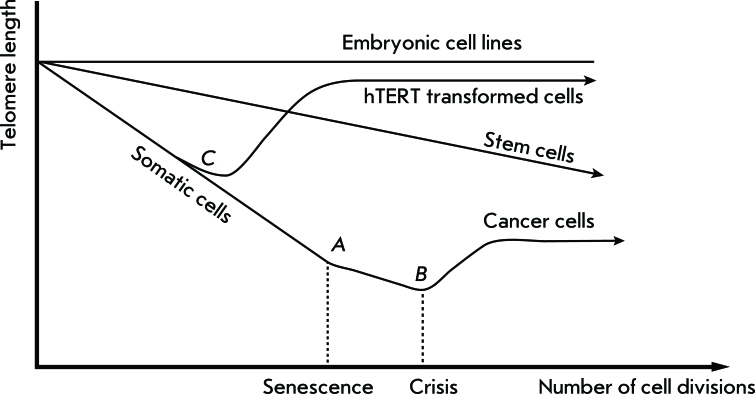
Dependence of telomere length on the number of cell divisions in different cell types: embryonic cell lines, somatic cells, hTERT-transformed cells. A– Hayflick limit, B – crisis with subsequent apoptosis or tumor transformation, C – cell transformation by *hTERT* gene.

Telomerase activity has not been detected in the vast majority of normal human cells; however, it manifests itself in reproductive organs and embryonal tissues. Thus, the enzyme is active in stem cells and some rapidly regenerating tissues, such as the intestinal epithelium; however, in this case telomerase activity is usually lower than it is in tumor cells ( *Fig. 2* ). Telomerase activity is more typical of malignant tumors, its activity and detection frequency being noticeably lower in benign tumors [10]. Detection of telomerase activity is used for both tumor diagnostics and in the search for potential anti-tumor drugs that would act as telomerase inhibitors. Furthermore, telomerase participates in tissue regeneration after the partial removal of the liver or cardiac infarction. In addition, the role of telomerase in the ageing process, in connection with the telomere’s function as a cell-timing mechanism, attracts a significant degree of interest.

##  METHODS COMPRISING THE AMPLIFICATION OF TELOMERASE-SYNTHESIZED DNA (TRAP) 

The most common methods for detecting telomerase activity are TRAPs (telomeric repeat amplification protocols) [11], which allow one to perform semi-quantitative and quantitative analyses, using some of their modifications. Such modifications include: increase of the analysis rate, replacement of the radioactive label by nonlabeled compounds, the reduction of the amount of side products, etc. Among these methods are the scintillation proximity assay, hybridization protection assay, transcription amplification assay, and the magnetic bead-based extraction assay [12]. Some modifications even enable to detect telomerase activity within a single cell [13].

**Table 1:** Some oligonucleotides that are used in different TRAP modifications

Oligo­nucleo­tide name	Oligonucleotide sequence
TS	5’-AATCCGTCGAGCAGAGTT-3’
CX	5’-(CCCTTA)_3_CCCTAA-3’
ACX	5’-GCGCGG(CTTACC)_3_CTAACC-3’
RP	5’-TAGAGCACAGCCTGTCCGTG-3’
RPC3	5’-TAGAGCACAGCCTGTCCGTG(CTAACC)_3_-3’
TSG4	5’-GGGATTGGGATTGGGATTGGGTT-3’

**Fig. 3:**
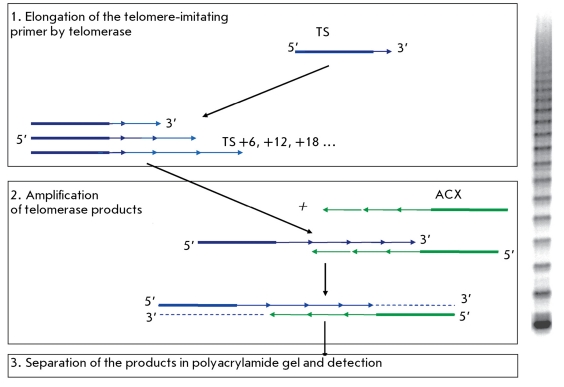
Telomeric repeat amplification protocol (TRAP).

The telomeric repeat amplification protocol can be subdivided into three main stages: primer elongation, amplification of telomerase-synthesized DNA, and thirdly, its detection. At the elongation stage, telomeric repeats are added to the telomere-imitating oligonucleotide (TS, *Table 1* ) by telomerase present in the cell extract. Then, PCR-amplification of telomerase-synthesized DNA is carried out with the use of specific primers (telomere-imitating and reverse primers). At this stage, different labels can be incorporated into the telomerase-synthesized DNA, such as radioactive, fluorescent, or affine labels. This stage is then followed by detection (in the original method, it comprises electrophoretic separation of PCR products and photographing) ( *Fig. 3* ).

The original TRAP method has several drawbacks. Initially, the CX oligonucleotide, which complementarily overlaps with TS for several bp, is used in the amplification of PCR products. It results in the dimerization of primers and products that emerge due to the interaction between primers. Even when using the optimal ACX primer with the noncomplementary TS end, a background signal may emerge during the analysis of concentrated extracts from tumor tissues [14]. An additional problem is encountered when using reverse primers which completely correspond to telomeric repeats. The primers are not annealed at the matrix edges during PCR (as a result of periodicity in telomeres), and hence false signals emerge. This problem is typically solved by adding regions that are noncomplementary to telomeres ( *Table 1* ) with a 5’-end nontelomeric “appendix” made of 6 bp to the primer ends. In order to reduce nonspecific signals, it is possible to use a combination of several primers that are used as reversed ones (more information on the two-primer system is given below, primers RP and RPC3 are given in *Table 1* ). Oligonucleotide TSG4 ( *Table 1* ) can also be added to the TRAP mixture in order to assess the effect of duplex-stabilizing inhibitors; this oligonucleotide does not require the synthesis of several repeats by telomerase before the inhibitor begins its action [15]. The advantages and drawbacks of the various nucleotides used in TRAP were discussed more thoroughly in [12]. Moreover, in the case when PCR is used for signal amplification, the PCR inhibitors contained in the specimen can impact the results of telomerase activity detection.

 Originally, in the TRAP method, PCR products were detected in polyacrylamide gel (PAAG) on account of the radioactive label, which was introduced using a radioactively labeled primer or incorporated into the DNA during the reaction. The method allows to perform a qualitative assessment of the activity and processivity of telomerase in cell and tissue extracts; however, as previously stated this requires radioactive specimens. 

PCR at the second stage of TRAP allows to obtain an amount of DNA sufficient for gel staining, e.g., using ethidium bromide [16] (an appreciably strong mutagen with a low sensitivity), silver nitrite [17] (its sensitivity is equal to that when using the radioactive label; however, the method is more laborious and relatively expensive), and Sybr Green [18] and its analogs (its sensitivity is equal to that when using the radioactive label [19], while mutagenicity is considerably higher than that in ethidium bromide, although it is an intercalating dye, as well). Fluorescent labeling of the nucleotides employed in TRAP can also be used [20].

**Purification of telomerase-synthesized DNA and TRAP efficiency**

 It is appreciably simple to obtain extracts of tumor cells or cell lines and detect telomerase activity in them, even though the tissues are composed of several cell types and may contain compounds that have an effect on the quantitative and even qualitative assessment of telomerase activity. Therefore, false-positive or false-negative results can be obtained, and they may affect the validity of the diagnosis and prognosis of a disease. The features of biopsic specimens, such as large volumes of fluid (blood, etc.) or presence of numerous normal cells, may complicate the detection of telomerase activity, as well. In these cases, the extraction of telomerase-synthesized DNA can be carried out using modified magnetic beads. During this procedure, PCR inhibitors are removed or strongly diluted. The TRAP method involving this extraction consists of three main stages: the elongation of the substrate-imitating oligonucleotide by telomerase, the extraction of telomerase-synthesized DNA using modified magnetic beads, and amplification. 

During the extraction stage, telomerase-synthesized DNA is hybridized with the C-rich biotin-conjugated primer (CCCTAA) _2_ and isolated from the extraction mixture using the magnetic beads coated with streptavidin-coated magnetic beads. Then, the telomerase-synthesized DNA is released from the complex by heating, and PCR is carried out. The sensitivity of this modification towards PCR inhibitors is lower by an order of magnitude as compared with the standard TRAP method; its efficiency is slightly higher during the analysis of tissue and other complex specimens [21]. Phenol-chloroform extraction can be used instead of the biotin-conjugated primer and magnetic beads [16], although in this case it becomes more difficult to remove the impurities that are more soluble in water than in organic solvents.

**Internal standards for TRAP**

Internal standards (amplified with the same primers as telomerase-synthesized DNA) allow for the presence of Taq polymerase inhibitors (e.g., gem-containing compounds) in the specimens to be taken into consideration, and for the performing of the total control of PCR. They can also be used for the standardization of the amount of telomerase-synthesized DNA. There are two most common standards, with the length of 36 and 150 bp. The 36 bp standard is excessively amplified if the specimens have a low telomerase activity, competes with the telomerase-synthesized DNA, and provides a false-negative signal [18]. The 150 bp standard is more sensitive towards the Taq polymerase inhibitors present in the reaction mixture. The standards can be used in TRAP with real-time PCR and primers in which the fluorescent label differs from that in primers for the amplification of telomerase-synthesized DNA [22].

**TRAP with an additional specific reverse primer (“two-primer” TRAP)**

**Fig. 4:**
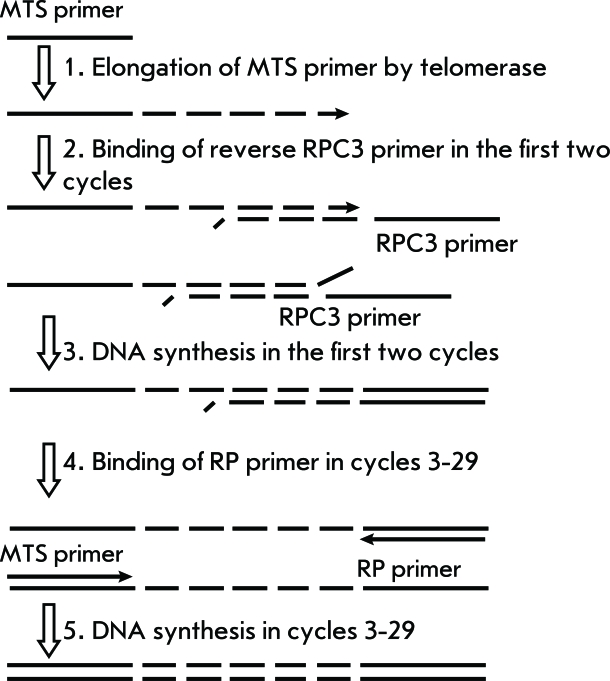
“Two-primer” TRAP scheme [23, 24].

Two-primer TRAP is a modification of the standard TRAP which is used to reduce false signals ( *Fig. 4* ).

In this TRAP version, no electrophoretic analysis of PCR products is carried out. Instead, the total radioactivity is assessed as a criterion of telomerase activity. The telomerase-synthesized DNA is amplified using two reverse primers with lengths of 20 (RP) and 38 (RPC3) nucleotides ( *Table 1* ), in the presence of [ ^3^ H]TTP or [α- ^32^ P]dСTP; the amount of the longer primer added is 50 times lower than that of the shorter primer. After the PCR, the two-chain DNA is isolated by deposition and filtration. The advantage of this method is that, due to the low concentration of the RPC3 primer, the amount of products of interaction between the primers is reduced, while the presence of standard amounts of the RP primer ensures a significant degree of amplification of the telomerase-synthesized DNA [23, 24]. This method makes it possible to qualitatively determine telomerase activity in extracts of tissues and cell lines. The sensitivity threshold of this method is the 10-cell extract of the telomerase-positive cell line.

**TRAP with fluorescence resonance energy transfer (FRET)**

**Fig. 5:**
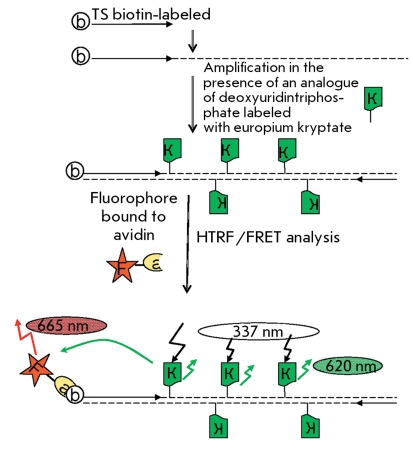
Scheme of the TRAP method with time-resolution fluorescence energy resonance transfer [26]. F – fluorophore, a – avidin, b –biotin, and K – analog of deoxyuridintriphosphate labeled with europium kryptate.

One of the alternatives to DNA staining in the TRAP method is to use primers with energy transfer (amplifluors). Amplifluors are characterized by their unique hairpin structure, which contains a donor (fluorescein) and acceptor (4’-dimethylaminophenyl-azobenzoic acid). Fluorescence manifests itself only in the case when a primer incorporates into PCR products; i.e., the necessity for using radioisotopes disappears, and the volume of post-PCR analysis considerably decreases. The replacement of the telomerase substrate and reverse primer by amplifluors allows to achieve an increasingly high intensity of the fluorescent signal. The primers that are used for amplification of the internal standards need to have a donor-acceptor pair with a fluorescence wavelength that differs from that of the primers used for amplification of telomerase-synthesized DNA [22].

The method involving the use of the time-resolution fluorescence resonance energy transfer (HTRF) combines the standard FRET technique and a long-lived fluorescent donor. The method is based on the use of europium or terbium cryptate complexes. These lanthanides are characterized by a long fluorescence decay period, whereas the complexation of macrocyclic compounds yielding cryptate complexes additionally enhances their stability [25]. A deoxyuridin triphosphate labeled with europium cryptate was used to assess the amount of DNA in TRAP with biotin-conjugated oligonucleotide TS. After the allophycocyanin–streptavidin, a conjugate was added and the FRET signal of DNA in TRAP was observed [26] ( *Fig. 5* ). This method allows a semi-quantitative detection of telomerase activity in tissue and cell line extracts. The sensitivity threshold of this method is the 10-cell extract of the telomerase-positive cell line.

**TRAP with detection using the scintillation proximity assay**

**Fig. 6:**
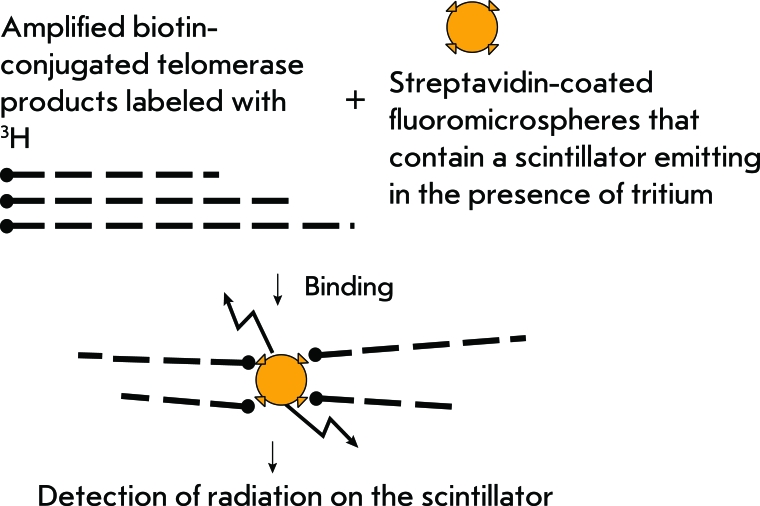
Scheme for TRAP with scintillation proximity assay.

Another means for the detection of the DNA amplified in TRAP without PAAG is the scintillation proximity assay. When used in combination with the traditional TRAP, it helps increase the rate of detection of telomerase activity. 5’-Biotin-conjugated oligonucleotides act as substrates in this method; the amplification takes place in the presence of [ ^3^ H]TTP. Biotin-conjugated ^3^ H-labeled DNA binds with streptavidin-coated fluoromicrospheres, which contain a scintillator that emits in the presence of tritium ( *Fig. 6* ). Thus, instead of separating DNA in a gel followed by photo-detection, the yield of PCR products is assessed on a scintillation counter, which allows to reduce the assay time after the PCR to 1 h and semi-quantitatively determine telomerase activity in large series of tissue and cell line extracts. The sensitivity threshold of this method is the 10-cell extract of the telomerase-positive cell line. The major drawback of this method is the use of tritium. Furthermore, like most methods for detecting TRAP products without PAAG, it is sensitive to PCR artifacts [27].

**TRAP with detection using the hybridization protection assay**

**Fig. 7:**
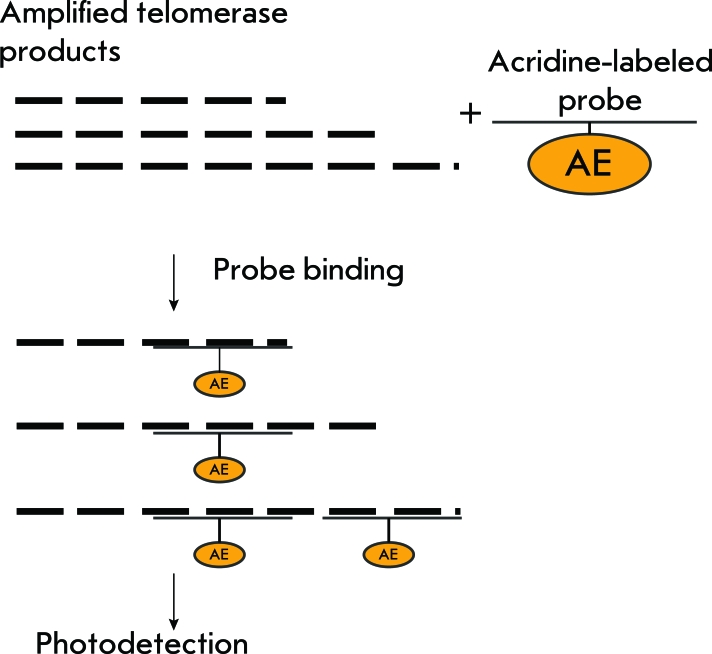
Scheme for TRAP with hybridization protection assay.

**Fig. 8:**
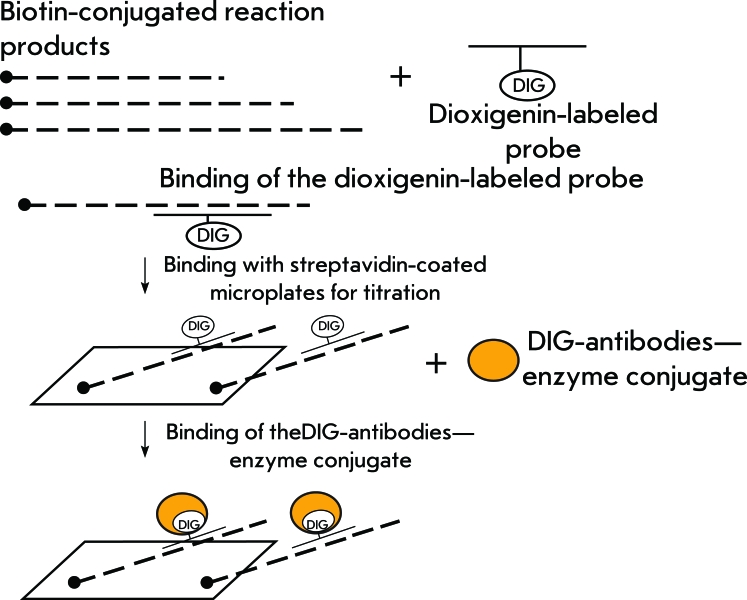
Scheme for ELISA detection of PCR products in TRAP-ELISA (or detection of telomerase reaction products in direct ELISA).

TRAP modification in which the hybridization protection assay (Hybridization protection assay-TRAP) is used is a safer modification. Probes labeled with covalently bound acridine [28] are used in this method for detecting DNA after the amplification ( *Fig. 7* ). The same detection scheme is used in the transcription scheme of telomerase repeat amplification. This method allows for semi-quantitative determination of telomerase activity in tissue and cell line extracts. The sensitivity threshold of this method is the 10-cell extract of the telomerase-positive cell line.

**TRAP combined with enzyme-linked immunosorbent assay (ELISA)**

In the TRAP-ELISA method, DNA after the amplification is determined colorimetrically, facilitating the qualitative and semi-quantitative assessment of telomerase activity. Biotin-conjugation of the TS primer allows for the binding of the amplified DNA to streptavidin-coated microplates ( *Fig. 8* ). The amplified DNA denatures and is hybridized with digoxigenin (DIG)-labeled probes, which demonstrate specificity towards telomeric repeats, and binds to the microplates due to the streptavidin–biotin interaction. This complex can be detected using polyclonal sheep antibodies to DIG conjugated to horseradish peroxidase; the activity of the latter is determined colorimetrically [29, 30]. This method differs from the hybridization version by the emergence of a second step of signal amplification due to the enzymatic reaction.

 One of the drawbacks of the TRAP-ELISA method is the complexity in separating the telomerase-positive and telomerase-negative controls, which may result from the absence of internal controls and two steps of signal amplification. Nevertheless, the TRAP-ELISA method is faster as compared with TRAP, which is based on the separation of the amplified DNA in gel. This fact makes it possible to use the TRAP-ELISA method in screening studies. The method is suitable for semi-quantitative determination of telomerase activity in tissue and cell line extracts. The sensitivity threshold of this method is the 10-cell extract of the telomerase-positive cell line. 

**TRAP with electrochemical detection**

After the PCR, the nonreacted nucleotide triphosphates can be separated, followed by treatment of the remaining products with 3 M HCl. In the method under consideration, dGMP, one of the products of the complete hydrolysis of the amplified DNA, is determined electro-chemically [31]. The method makes it possible to semi-quantitatively determine telomerase activity in tissue and cell line extracts. The sensitivity threshold of this method is the 10-cell extract of the telomerase-positive cell line. The method has no special advantages over other methods in which no gel-electrophoresis or radioactive labeling are used. However, the method is more laborious.

**TRAP with real-time PCR**

Real-time PCR is used for simultaneous DNA amplification and measurement of the amount of products obtained after each amplification cycle. Standard TRAP, combined with real-time PCR, allows to obtain quantitative results [32, 33]. This method is suitable for studying telomerase inhibitors and analyzing large specimen series [12]. When comparing it with the conventional TRAP, it can be noted that over-estimation of telomerase activity and leveling of small differences in the activity is possible without the assessment of the amount of real-time PCR products, due to saturation of the PCR reaction in the final stages. Moreover, the possibility of dimerization of primers resulting in the emergence of a false-positive signal causes an additional problem. This problem can be made more acute when using the reverse anchor-primer, which will impede dimerization, reduce the number of primers and number of cycles, resulting in dimerization of primers to occur mostly at late stages of the program (35–39 cycles); in the meantime, the number of cycles is 28 in the standard TRAP. This problem is also mitigated when measuring fluorescence in the early exponential phase of PCR [32]. The method makes it possible to quantitatively determine telomerase activity in tissue and cell line extracts. The sensitivity threshold of this method is 10 to 50 cell extracts of the telomerase-positive cell line.

Similar to the standard TRAP with gel-electrophoresis, TRAP with real-time PCR has several variants of detection, e.g., using the probes which are specific to the telomerase-synthesized DNA amplified in the PCR and fluoresce only as a component of a duplex. Otherwise, these probes will form the nonfluorescent hairpin structure [34]. The advantages of this method include a high rate of detection of telomerase-synthesized DNA upon flow analysis and enhanced specificity due to the use of corresponding probes [35]. The method allows semi-quantitative determination of telomerase activity in tissue and cell line extracts. The sensitivity threshold of this method is the 10-cell extract of the telomerase-positive cell line.

**TRAP on microchips**

 TRAP on microchips is the combination of the two-primer TRAP and binding of PCR products on chips, followed by probe hybridization and detection. Various fluorescent labels are used to determine telomerase-synthesized DNA and the internal standard; e.g., Cy3 for the telomerase-synthesized DNA being amplified and Cy5, for the standard. 

*In situ* **TRAP**

Telomerase activity is typically analyzed in a cell extract. If cell lines are homogeneous, the result represents the telomerase activity in cells. When the extracts of tumor tissue specimens are investigated, it is reasonable to consider only the mere fact of the presence or absence of telomerase activity, since the content of tumor cells in such specimens varies from 3 to 90% and more. In this case, the results obtained reveal nothing about the level of telomerase activity in various types of cells constituting the tissue or on the differences between tumor and normal cells within the same tissue (e.g., an increase in telomerase activity in blood is associated with the emergence of activated leukocytes or degeneration of certain blood cells). In order to overcome this drawback, the *in situ* TRAP method was designed, in which FITC-labeled direct and reverse primers are used [36]. In this method, the cell suspension specimens are immobilized onto silane-coated glasses and dried with cold air. The reaction mixture for TRAP is then applied onto each specimen, followed by incubation. Telomerase is inactivated via heating, the reverse primer with polymerase is added, and *in situ* PCR is performed. Upon completion of the reaction, the glasses are analyzed on a fluorescence microscope; cell types are identified by the staining procedure. The fluorescence intensity and its localization (nucleus/cytoplasm) are used to determine telomerase activity in separate cell types within the mixture. High telomerase activity in urogenital and bronchial lavages manifests itself through bright fluorescence of the nuclei of malignant (but not benign) cells [37, 38]. A comparison with the conventionally stained specimens points to association between the fluorescence in the nucleus with malignant cells, whereas the signal from cytoplasm is detected in granulocytes and macrophages [39]. A similar correlation was found during the analysis of pleural effusion [40]. Moreover, *in situ* TRAP has been successfully used not only for the cell suspension specimens, but also on tissue sections (in particular, for diagnostics of liver cancer) [41]. The method allows for a semi-quantitative determination of telomerase activity and its localization in isolated cells of tissues and cell suspensions.

**Transcription amplification of telomerase-synthesized DNA**

**Fig. 9:**
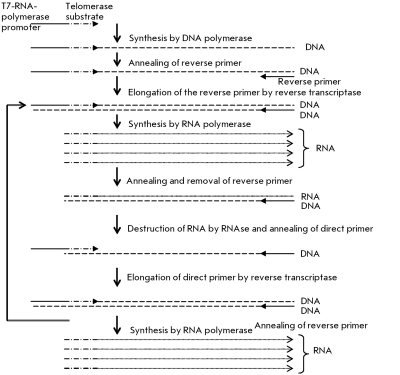
Scheme of telomerase activity detection with the PCR amplification of telomerase products for transcriptional amplification [12, 42].

In order to increase the amount of telomerase-synthesized DNA, PCR is replaced by transcription amplification. Combined with “hybridization protection,” this method allows to determine the telomerase activity as quickly as after 4 hours. The primer, which acts as a substrate for telomerase, contains the site of T7-RNA-polymerase binding, along with the telomere-imitating sequence. The telomerase-synthesized DNA acts as a substrate for hybridization of the reverse primer, which contains an additional sequence that is noncomplementary to the telomeric repeats. Due to this sequence, the reverse primer binds with the end of the telomerase-synthesized DNA, and the completion of the double-chain DNA takes place at the next stage. T7-RNA-polymerase uses this DNA as a matrix to synthesize RNA. This RNA is used by the reverse transcriptase to synthesize a new DNA chain. RNA is then removed by RNase; the second DNA chain of the single-chain DNA is completed with the aid of the direct primer ( *Fig. 9* ).

Using this scheme, telomerase-synthesized DNA can be amplified at 40 ^o^ С for 75 min in amounts comparable with PCR. The subsequent reactions may be carried out in the same tube as those in which amplification takes place. The method’s sensitivity allows to detect telomerase activity in specimens consisting of 1–1,000 cells. The method is sensitive towards the presence of RNases [12, 42]. The major advantage of this method over TRAP with PCR amplification is that no heating of a specimen is required upon amplification and that the specific Taq polymerase inhibitors are neglected. This method makes it possible to semi-quantitatively determine the telomerase activity in tissue and cell line extracts. The sensitivity threshold of this method is the 10-cell extract of a telomerase-positive cell line.

##  DIRECT DETECTION OF TELOMERASE-SYNTHESIZED DNA 

**Detection of telomerase activity using direct incorporation of a radioactively labeled substrate**

The method is based on the ability of telomerase to elongate oligonucleotides in the presence of dNTP. If an oligonucleotide or one of the dNTP (typically, dGTPl, since telomerase-synthesized telomeres are enriched in guanine residues) contains a radioactive label, telomerase activity can be electrophoretically assessed by its incorporation into DNA [43]. The major drawbacks of this method include the use of large amounts of radioactive isotopes and insufficient sensitivity (in many cases), which is conditioned by a low telomerase content in cells and tissues.

 The first method used for telomerase activity detection was a method in which radioactive labeling was used without any additional amplification. This method is still employed today for the thorough investigation of telomerase-synthesized DNA and quantitative measurements. The method allows for qualitative determination of the activity and processivity of telomerase in cell line extracts; however, it is characterized by low sensitivity (10,000 cells of telomerase-positive cell line). 

**Determination of telomerase-synthesized dna by changes in surface plasmon resonance**

**Fig. 10:**
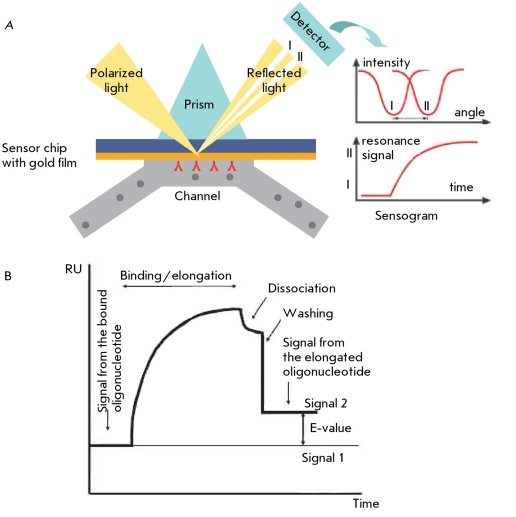
Scheme of the use of surface plasmon resonance (SPR) for detecting macromolecules; (A) sensogram corresponding to the general scheme and (B) SPR sensogram for telomerase activity detection. RU – resonance units. The difference between signals 1 and 2 represents DNA that was synthesized by telomerase [44].

Surface plasmon resonance (SPR) manifests on a metal’s surface under conditions of total internal reflection and is characterized by a nonspecific angle of reflection and, therefore, the refraction index. As this effect manifests itself on the surface of a metal film, it propagates deep into the solution with exponential damping as a distance function. The interactions between the molecules change the damped wave, which has an effect on the characteristics of the surface plasmon and manifests itself in the variation of the resonance angle and the refraction index in the near-surface layer. The interaction between bio-molecules is judged by the alteration of the refraction index ( *Fig. 10A* ).

 SPR can be used to determine telomerase activity as the corresponding elongation of a telomere-imitating oligonucleotide, using a biosensor. 

5’-Biotin-conjugated oligonucleotides, which contain telomeric repeats, are *in situ* immobilized on the surface of a dextrane sensor pretreated with streptavidin on a BIACORE instrument. They are then treated with a cell extract. If the extract contains telomerase, binding of telomerase takes place and the oligonucleotide is elongated, which is determined by SPR after proteins are removed with a sodium dodecyl sulfate solution ( *Fig. 10B* ). The degree of oligonucleotide elongation depends on specimen concentration and reaction time. The results of the analysis of tumor and cell line extracts using this method, as well as the results of testing telomerase inhibitors, correlate with the data obtained by the TRAP method [44]. The signal intensity can be increased by treating the sensor with telomerase-synthesized DNA with antisense oligonucleotides with respect to the telomeres that are covalently bound with streptavidin and gold nanoparticles [45].

 This method allows to quantitatively determine telomerase activity in tissue and cell line extracts and collect data on the kinetics of the reaction, demonstrating the binding and dissociation of telomerase from the substrate. The sensitivity threshold of this method is the 50-cell extract of the telomerase-positive cell line. 

**Determination of telomerase activity using oligo-modified magnetic particles and NMR**

**Fig. 11:**
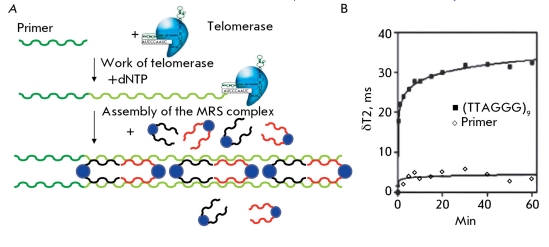
Telomerase magnetic nanosensor. A - Complex assembly between the synthesized telomere repeats and oligonucleotide magnetic nanoparticles conjugated with antitelomere. B - Induced magnetic T2 changes as a function of time after adding an oligonucleotide consisting of either a 54-bp telomeric repeat or the primer [46].

This method is based on the use of magnetic particles of an iron oxide which are modified with oligonucleotides that are complementary to telomeric repeats. These particles bind to the telomerase-synthesized repeats due to complementary interactions and form extended linear structures (MRS complexes) ( *Fig. 11A* ). The effect of this hybridization is determined by the spin-spin relaxation time. During the formation of an organized nanoparticle ensemble, there is a noticeable change in the magnetic relaxation time of the surrounding water molecules, which can be measured in a relaxometer (the relaxometer measures both the spin-spin relaxation times of the nuclear magnetization of proton-bearing liquids in multi-component systems and the content of components with different relaxation times). The magnetic switching occurs rapidly, attaining half of the maximum change after 30s and reaching a plateau after 40–60 min ( *Fig. 11B* ). The magnetic switching has been confirmed by magnetic force microscopy and by correlation of the magnetic switching and the size of the nanoensembles being formed. The local distortion of the magnetic field increases on nanoparticles in ordered ensembles, whereas the nonordered nanoparticles provide a considerably lower magnetic effect [46].

The relaxation time was measured in a volume of 200–500 µl in an NMR spectrometer at a magnetic induction of 0.47 T. For the magnetic resonance scanning of 384 well plates with 50 µl of the mixture at 1.5 T, the spin-spin relaxation (T2) and spin-lattice relaxation (T1) times were assessed. The adaptation to the flow treatment using magnetic resonance scanning made it possible to analyze several hundred specimens as quickly as in several tens of minutes with high sensitivity [46]. The method allows for the quantitative determination of telomerase activity in tissue and cell line extracts using the high-performance microplate format. In addition to the common instruments and reagents, these analyses require a plate NMR-spectrometer and a specimen of oligonucleotide-modified nanoparticles. The sensitivity threshold of this method is the 10-cell extract of the telomerase-positive cell line.

**Determination of telomerase activity using the quartz crystal microbalance technique**

 An Au-quartz resonator can be used for a microgravitometrical analysis of telomerase activity, according to the quartz crystal microbalance technique. Quartz crystals possess a piezoelectric effect. Alternation of the current imposition results in the emergence of oscillations in a quartz crystal; in specially curved crystals, a current of a certain frequency may lead to the formation of a stationary wave. Its resonance frequency can be quite accurately determined. The wave changes upon ligand binding on the crystal’s surface. The quartz crystal microbalance technique in liquids is utilized to determine the affine binding of molecules (in particular, proteins) on surfaces that contain the corresponding recognition sites. 

**Fig. 12:**
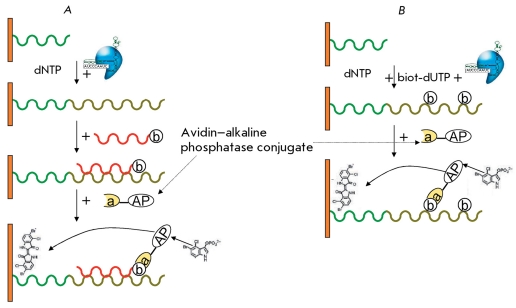
Processes on the sensor in quartz crystal microbalance and electrochemical methods. A - Insertion of a biotin-labeled dUTP into the telomerase-synthesized DNA. B - Hybridization of biotin-labeled oligonucleotide with telomerase-synthesized DNA [47]. b – biotin as a component of the oligonucleotide, a-AP –avidin–alkaline phosphatase conjugate, dUTP – mixture of nucleoside triphosphates, biot-dUTP – biotin-labeled dUTP.

In this method, the telomere-imitating oligonucleotide is bound to the sensor’s surface, elongated by telomerase. Next, the synthesized DNA chain is hybridized with a biotin-conjugated oligonucleotide ( *Fig. 12A* ). An alternative variant includes the elongation of the telomere-imitating oligonucleotide that is bound to the sensor in the presence of a biotin-labeled NTP ( *Fig. 12B* ). Next, the sensor’s surface is treated with an avidin–alkaline phosphatase conjugate and a solution of alkaline phosphatase substrate – 5-bromo-4-chloro-3-indolylphosphate, which upon hydrolysis yields an insoluble product on the sensor’s surface. When determining telomerase activity through a decrease in the resonance frequency of the crystal, it is possible to observe the steps of oligonucleotide elongation, binding with the telomerase-synthesized DNA of the avidin–alkaline phosphatase conjugate, and the deposition of products of the reaction catalyzed by the alkaline phosphatase on the crystal’s surface [47]. The method allows for the quantitative determination of the telomerase activity in tissue and cell line extracts. The sensitivity threshold of this method is the 3,300-cell extracts of the telomerase-positive cell line at a high rate. A frequency analyzer and an Au-quartz crystal are needed in this method. Moreover, the identification of artifact signals is complicated.

**Electrochemical detection of telomerase activity with ferrocenyl naphthalene diimide**

**Fig. 13:**
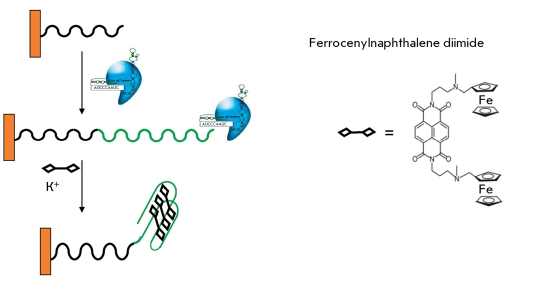
Assembly on the electrochemically detectable complex upon telomerase activity detection with ferrocenylnaphthalene diimide [48].

This method is based on the ability of telomeric repeats to form G-quadruplexes. Telomerase elongates an oligonucleotide that is immobilized on an electrode. The synthesized telomeric then repeats when there is a high concentration of potassium ions, forming quadruplexes; while ferrocenyl naphthalene diimide can stoichiometrically bind with DNA quadruplexes under these conditions and stabilize their structure ( *Fig. 13* ). In turn, the bound ferrocenyl naphthalene diimide can be detected using electrochemical methods. In this method, the quantities of DNA fragments that are folded into a quadruplex are assessed. This number depends not only on the total number of repeats, but also, to an unpredictable degree, on the length and position of individual DNA fragments, which can be considered a drawback. The sensitivity of the method is sufficient to detect the telomerase-synthesized DNA without amplification [48] (100–1,000 cells of the telomerase-positive cell line); however, this method has not as yet been tested on a tumor tissue extract.

**Biobarcode assay for telomerase activity detection**

**Fig. 14:**
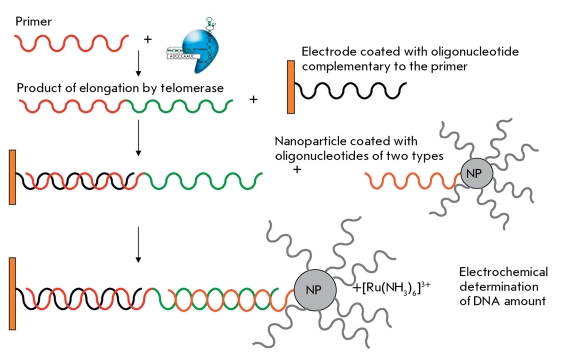
Biobarcode-based electrochemical telomerase detection [49]. NP – nanoparticles.

Today, the most sensitive method for direct detection of telomerase activity without amplification of the telomerase-synthesized DNA is based on the biobarcode system. In the original biobarcode system, magnetic particles bind to a target, which in turn binds to nanoparticles covalently modified with an oligonucleotide due to antigen-antibody interactions. These nanoparticles serve as a biological barcode. The resulting “sandwich” is isolated from the reaction mixture in the magnetic field and denatured; the result is determined by the released DNA fragments. In the system of telomerase activity detection, the telomerase-synthesized DNA is recognized using complementary DNA fragments. DNA nanoparticles consist of gold nanoparticles and oligonucleotides of two types; one of these can form a duplex with the telomerase-synthesized DNA, whereas the second cannot. Because of this, the probability of binding another DNA-target to the same nanoparticle is reduced ( *Fig. 14* ). The electroactive complex [Ru(NH _3_ ) _6_ ] ^3+^ , which is capable of binding to negatively charged DNA chains due to electrostatic interactions, is used for detection [49]. The method allows to quantitatively detect telomerase activity in cell line extracts; however, it has not been tested on tumor tissue extracts yet. The sensitivity threshold of this method is the 10-cell extract of the telomerase-positive cell line.

**Telomerase activity detection using optical biosensors**

**Fig. 15:**
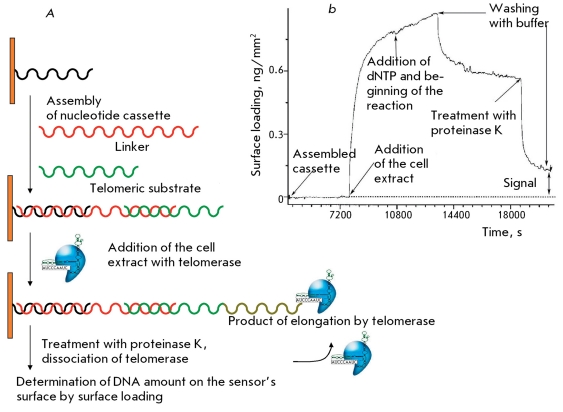
A - The scheme of formation of telomerase-synthesized DNA on the sensor surface. B - Sensogram for determination of the surface loading on the optical sensor [50].

The principle of this method is to a certain extent similar to that of SPR. The method is based on the fact that upon binding of a target, the refraction index on the sensor’s surface changes in proportion to the amount of bound targets. A cassette consisting of three oligonucleotides helps to avoid steric impediments. Phosphate groups covalently interact with the surface via the 5’-end of an oligonucleotide. Then, an oligonucleotide containing a short noncomplementary region on its 3’-end complementarily binds to the immobilized DNA. The prominent 3’-end of the DNA is modified with phosphorothioate, which enhances the affinity of telomerase-primer binding by a factor of 10 [50]. After treatment with a telomerase-containing extract, the enzyme is removed from the sensor’s surface by proteinase K in the presence of dNTP ( *Fig. 15A* ).

Telomerase activity was assessed in this case by the sensogram of the surface charge (ng/mm ^2^ , determined by the change in the refraction index) ( *Fig. 15B* ). The method allows to quantitatively detect the telomerase activity in cell line extracts; however, it cannot be used for tumor tissue extracts, since its sensitivity threshold is the 10 ^5^ -cell extract of the telomerase-positive cell line. In addition, phosphorothioate-modified oligonucleotides and a special optosensor are used in this method.

**The system of telomerase activity detection based on quantum dots**

A quantum dot is a conductor or semiconductor fragment that is limited along all three spacial dimensions and contains conductivity electrons. The dot has to be quite small in order to produce considerable quantum effects. This can be achieved if the kinetic energy of an electron *E*  = *ћ ^2^ /md* ( *d* is the characteristic dimension of a dot, *m * is the effective mass of an electron on the dot) conditioned by the uncertainty of its momentum is considerably higher than that of all other energy scales, primarily, higher than the temperature expressed in energy units. Any appreciably small piece of the metal or semiconductor can serve as a quantum dot. In this microcrystal, an electron feels as if in a three-dimensional potential well; it has many bound energy levels with a characteristic distance between them (the accurate expression for the energy levels depends on the shape of a dot). Like upon transition between the energy levels of an atom, a photon can be emitted upon transition between the energy levels of a quantum dot. Unlike in atoms, the transition frequencies can be easily controlled by varying the crystal’s dimensions.

**Fig. 16:**
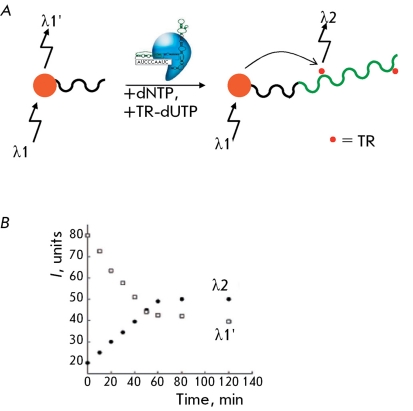
A - Telomerization of the oligonucleotide bound with CdSe-ZnS by a quantum dot with the incorporation of Texas Red-labeled dUTP. B - Switching from the wavelength of quantum dot fluorescence (λ1) to Texas Red fluorescence (λ2) upon telomerization of the nucleotide bound with CdSe-ZnS by a quantum dot with the incorporation of TR-dUTP [51]. TR – Texas Red.

A telomere-imitating oligonucleotide modified at its end with the thio group is attached to a nanoparticle (quantum dot). This quantum dot is capable of fluorescing by absorbing a quantum with a wavelength of λ1 (400 nm) and emitting a quantum with a wavelength of λ1’ (560 nm). If a modified fluorescent oligonucleotide TR-dUTP (dUTP labeled with Texas Red) is incorporated into DNA upon telomerase-elongation of an oligonucleotide attached to a quantum dot, a fluorescence energy transfer occurs ( *Fig. 16A* ), which is accompanied by a decrease in emission with a wavelength of λ1’ and the beginning of emission with a wavelength of λ2 (610 nm) ( *Fig. 16B* ). The method allows for a quantitative assessment of the telomerase activity. The sensitivity threshold is approximately 10,000 HeLa cells [51], which is not enough for an analysis of clinical materials.

Instead of incorporating TR-dUTP, it is possible to use the ability of telomeric repeats to fold into G-quadruplexes and bind hemin. In this case, an energy transfer takes place between a quantum dot and the G-quadruplex–hemin complex with corresponding fluorescence quenching. The sensitivity of this method is 270 T293 cells [52].

**Telomerase activity detection using an on-chip nanowire sensor**

 A sensor chip is a transistor comprising antibody-coated silicon nanowires with aldehydes groups on their surface, to which monoclonal antibodies can be linked. The conductivity of the antibody-coated nanowire varies depending upon the binding of antigens, which is detected. 

When using a sensor chip to determine telomerase activity, the transistor nanowire is modified by telomere-imitating oligonucleotides instead of antibodies. After the introduction of telomerase and dNTP, the oligonucleotides bound on the surface elongate, which results in change in the conductivity of the transistor to which the oligonucleotides are bound [53]. The sensitivity of this method is 100 HeLa cells.

A similar method is based on using an ion-selective field-effect transistor. In the presence of certain chemical agents, a potential emerges on the shutter of these transistors. It opens the conductivity channel of the transistor; i.e., current starts flowing through it. The current’s intensity is proportional to the concentration of the desired component. The Al _2_ O _3_ -shutter of the transistor is modified with a telomerase substrate. As a primer is elongated by telomerase, the potential on the shutter also changes. The method allows to determine telomerase activity in cell line extracts (this method has not been tested on clinical materials). Its sensitivity is 65 T293 cells [54]. The major advantage of the method is that a large number of different analyses of one specimen can be carried out on a single chip, which can be a set of sensors with respect to various markers. Moreover, the stages of telomerase binding and dissociation can be observed.

**Bioluminescence method to determine telomerase activity**

**Fig. 17:**
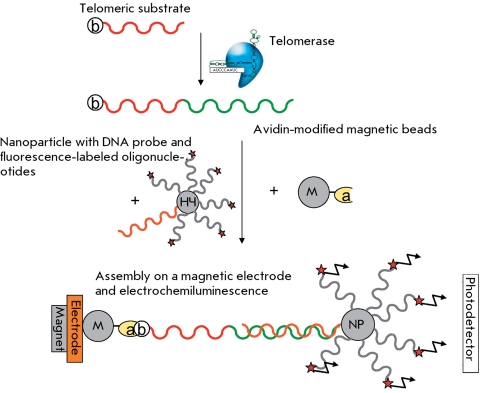
Electrochemiluminescence method for telomerase activity detection [54].

Determination of telomerase activity using the bioluminescence method is based on the fact that the telomerase-catalyzed elongation of the telomere-imitating oligonucleotide is accompanied by the cleavage of pyrophosphate; its amount is determined luminometrically. In the presence of adenosine-5’-phosphosulfate, ATP-sulfurylase converts pyrophosphate into ATP; its content is determined using the luciferin/luciferase method. According to [55], the sensitivity and specificity of the method is comparable with the sensitivity of TRAP-ELISA. This method allows quantitative determination of the telomerase activity in cell line and tissue extracts (having been tested on lung tumors). The advantage of the method is the linear dependence of the signal on the amount of telomerase-synthesized DNA, combined with high efficiency.

**Electrochemiluminescence method to determine telomerase activity**

Telomerase activity can also be determined by electrochemiluminescence (luminescence upon electrolysis). In this method, the 5’-biotin-conjugated primer is elongated by telomerase followed by incubation with a suspension of magnetic beads modified with avidin. The DNA immobilized on magnetic beads is hybridized with an electrochemiluminescence sample (modified with a ruthenium-bis(2,2’-bipyridine)(2,2’-bipyridine-4,4’-bicarbonate)-N-hydroxysuccinimide ester oligonucleotide that is covalently bound to a gold nanoparticle). These ternary complexes (magnetic bead–telomeric sample–electroluminescence sample) are washed and injected into a reaction cuvette in which they are magnetized to the working electrode and begin to luminesce under voltage ( *Fig. 17* ). This method allows for the quantitative determination of telomerase activity in samples that contain at least 500 HeLa cells [56]. It provides an appreciably high signal/noise ratio due to the stage of magnetic bead extraction; however, it has not been tested on clinical materials.

**Telomerase activity detection by FRET and total internal reflection fluorescence microscopy**

The FRET-based method is intended to distinguish the single-letter synthesis (a nonprocessive method of synthesis) and the beginning of synthesis of the second DNA repeat (a conditionally processive method of synthesis) by individual complexes of *Tetrahymena thermophila* telomerase. FRET was determined using the total internal reflection fluorescence microscopy that is based on the phenomenon of reflection of electromagnetic waves from the interface of two transparent media. The transparent media emerge under the condition that a wave comes from the medium with a higher refraction index, at an angle that exceeds the angle of total reflection. The intensity of the radiation penetrating into the second medium decreases in accordance with the exponential law, which allows to reveal the fluorescent objects that are excited by this radiation within an ~100-nm thick boundary layer with a resolution of up to 10 nm.

**Fig. 18:**
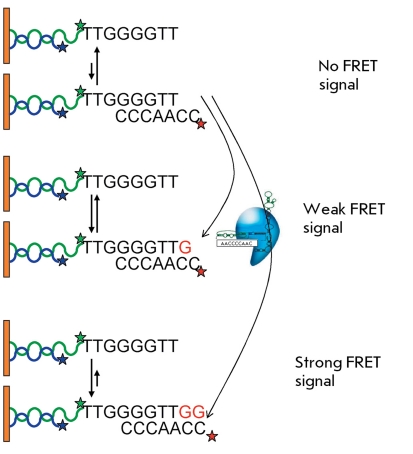
Telomerase activity detection using FRET and total internal reflection fluorescence [57].

Biotin-conjugated primers (TG) _8_ T _2_ G _4_ T _2 _ were bound on streptavidin-coated quartz slides and treated with a telomerase-containing extract in the presence of dGTP and ddTTP; the latter ceasing the synthesis at the second repeat. As a result, detectable products of two types were formed. The slides with the reaction products were treated with a Cy3-labeled oligonucleotide (Cy3-(CA) _8_ ) in order to stain each primer bound to the slide with Cy3 because of complementary interaction with a nontelomeric region of the oligonucleotide (TG) _8_ . Then, the system was treated with a Cy5-labeled detecting oligonucleotide (Cy5-C _2_ A _2_ C _3_ ), which was complementary to the telomeric sequence. This nucleotide can be more effectively bound with the longer telomerase product. The FRET signal is recorded between Cy3 and Cy5 [57] ( *Fig. 18* ). The method allows to identify individual signals from the elongation of the primer by telomerase.

In order to verify and normalize the system, primers of three types labeled with Alexa Fluor-488 were used: those corresponding to the sites without synthesis; to single-letter synthesis; and to the synthesis of the second repeat ((TG) _8_ T _2_ G _4_ T _2_ , (TG) _8_ T _2_ G _4_ T _2_ G, (TG) _8_ T _2_ G _4_ T _2_ G _4_ T) with 5’-end biotin, which were bound with streptavidin-coated quartz slides. Fluorophore Alexa Fluor-488 makes it possible to determine the density and localization of the primers bound to the slide [57].

The determination of telomerase activity using FRET and total internal reflection fluorescence microscopy allows to detect the elongation of individual primers by telomerase and can be combined with FRET-based methods of investigation of the telomerase structure. This method can be used to determine only the first 1.5 telomerase-synthesized repeats; i.e., it does not reveal total telomerase activity. The method has been tested only on *T. thermophila* telomerase and has been aimed only at solving research problems so far.

##  DETERMINATION OF TELOMERASE ACTIVITY BY ELISA SIGNAL AMPLIFICATION 

**Determination of telomerase activity using digoxigenin-labeled oligonucleotides complementary to telomeres**

The determination of telomerase-synthesized DNA by ELISA is appealing, since it is an isotope-free method. This method is based on hybridization of telomerase-synthesized DNA with a digoxigenin-labeled oligonucleotide and determination of the resulting complex by ELISA, using a dioxigenin antibody–alkaline phosphatase conjugate ( *Fig. 8* ). The sensitivity threshold of the method is 10 amol of the product, which is typical for digoxigenin systems. When using T293 cell extracts, the sensitivity level was 37,500 cells [58], which is insufficient for the analysis of clinical materials. The method for determining telomerase-synthesized DNA by ELISA is far from being the most sensitive; however, it has been completely automated and optimized for the search for telomerase inhibitors.

**Electrochemical determination of telomerase activity using avidin­–alkaline phosphatase conjugates**

This method is based on ELISA signal amplification with electrochemical detection. A thiolized oligonucleotide substrate, which is covalently attached to a gold electrode, is elongated by telomerase in the presence of NTP. ELISA is then carried out (similar to the method in which a quartz crystal microbalance is applied), yielding an insoluble product on the electrode’s surface, which is detected electrochemically ( *Fig. 12* ). Chronopotentiometry (the method based on measuring the variation of the electrode potential *Е * in time under a controlled (constant) value of the electrolysis current) is used for the detection. The larger the deposit on the electrode’s surface, the stronger the variation of the potential. This method allows for the quantitative determination of telomerase activity in tissue and cell line extracts at a high rate [47]. The sensitivity of this method is sufficient for determining telomerase activity in an extract corresponding to 1,000 HeLa cells.

**Determination of telomerase activity using a fluorimetric optosensor**

**Fig. 19:**
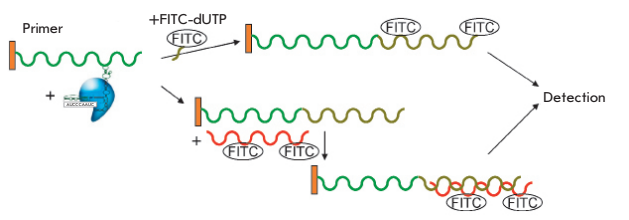
Scheme of formation of fluorescence-labeled telomerase-synthesized DNA detection by a fluorimetric optical sensor [59].

This optical sensor is based on fluorimetry (in fact, it is a fluorimeter with an optical fiber used in the excitation and detection systems). The oligonucleotide, the telomerase substrate, is modified at the 3’-end by phosphotioate in order to enhance the affinity of the telomere-oligonucleotide binding region of hTERT and, therefore, the rate of the telomerase reaction. This primer covalently binds to the surface of an optical biosensor; its elongation by telomerase is traced in real time by incorporation of dUTP labeled with a isothiocyanate fluorescein derivative (FITC), or the elongated chain is determined by hybridization of the FITC-labeled complementary DNA sample ((CCAATC) _4_ -FITC) ( *Fig. 19* ). No PCR amplification or additional purification stages are required in this method [59]. Furthermore, the efficiency of the telomerase function was compared in the presence of regular dNTP and in the presence of FITC-labeled dUTP. In order to achieve this, telomerase-synthesized DNA, in the presence of FITC-labeled dUTP, was labeled with a FITC-labeled complementary DNA sample. It turned out that telomerase was less efficient in the presence of FITC-labeled dUTP [59]. This method allows for the quantitative determination of telomerase activity; however, its sensitivity does not exceed 10 ^6^ –10 ^7^ , which is insufficient for analyzing clinical materials. In addition, this method uses the custom-built system of fluorescence detection.

**Determination of telomerase activity using DNAzymes**

 DNAzymes are DNA fragments with enzymatic activity. 

**Fig. 20:**
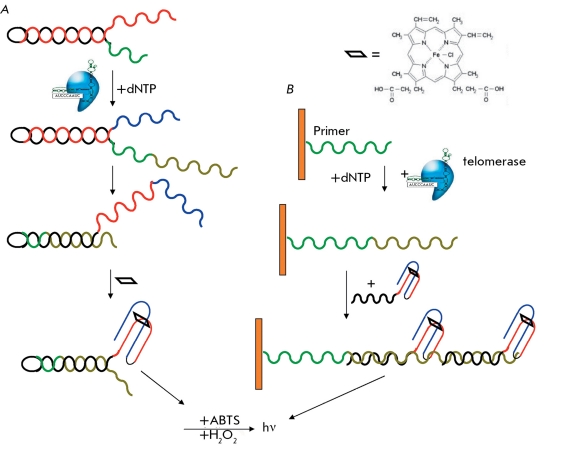
(A, B) Two schemes for telomerase activity detection with DNAenzymes [60, 61].

There are several ways in which DNAzymes can be used when analyzing telomerase. First, a telomerase substrate may have a hairpin at its 5’-end, which refolds as the substrate elongates, thus forming a catalytic structure ( *Fig. 20A* ). In doing so, one chain of the original hairpin forms a duplex with the *de novo * synthesized telomeric repeats, while the second hairpin in the presence of a hemin molecule forms a quadruplex with an incorporated hemin molecule with peroxidase activity. This complex catalyzes the oxidation of 2,2’-azino-bis(3-ethylbenzthiazoline)-6-sulfonic acid (ABTS) in the presence of H _2_ O _2_ , which results in the accumulation of a colored product ( *Fig. 20* ). The sensitivity of this method is approximately 10,000 HeLa cells, with a duration of the analysis of approximately 8 min [60]. The slow change in coloration of the extract from the control cells after the thermal treatment should be noted. These authors believe that this is associated with the nonspecific hemin sorption on the reaction mixture components. This method represents the number of elongated substrate molecules; however, it does not take into account the length of the chain synthesized by telomerase.

**Table 2:** Brief comparison of the methods for telomerase activity detection

Method for telomerase activity detection	Sensitivity*	Advantages	Drawbacks**	Reference
Direct incorporation of a radioactively labeled substrate	10^5^–10^6^	The absence of artifacts associated with PCR. The telomerase-synthesized DNA can be immediately observed in the gel. Its size and amount can be estimated.	Low sensitivity, the necessity of working with large amounts of a radioactive label, long-term exposition.	[43]
Determination of telomerase-synthesized DNA by the changes in surface plasmon resonance	20–100	The absence of artifacts associated with PCR. No radioactive label and PAAG. Information on the reaction’s kinetics. The possibility of signal detection against 1,000-fold excess of telomerase-negative cells. Telomerase binding and dissociation can be observed.	The BIACORE system and biotin-conjugated primers are required.	[44, 54]
Using oligo-modified magnetic particles and NMR	10	The absence of artifacts associated with PCR. No radioactive label and PAAG. A very high performance microplate format.	A NMR spectrometer and a sample of nanoparticles covalently bound to oligonucleotides are required.	[46]
Using the quartz crystal microbalance technique	3300	The absence of artifacts associated with PCR. High sensitivity. Rapid procedure.	A frequency analyzer and a Au-quartz crystal are required. Identification of artifact signals (in case of emergence) is difficult.	[47]
Electrochemically, using ferrocenyl naphthalene diimide	100–1000	The absence of artifacts associated with PCR. High sensitivity. Rapid procedure.	The number of structures folded into the quadruplex is assessed, which depends not only on the total number of repeats, but also is unpredictably dependent on the length and position of individual DNA fragments.	[48]
Using a biobarcode	10	One of the most sensitive methods today for direct telomerase activity detection without amplification of the telomerase-synthesized DNA	A modified electrode and nanoparticles coated with oligonucleotides of two types are required.	[49]
Using optical biosensors	10^5^	No radioactivity, no PAAG, no PCR.	Phosphorothioate-modified oligonucleotides and a special optosensor are required. The sensitivity is insufficient for clinical materials.	[50]
Based on quantum dots	10,000/270	No radioactivity, no PAAG, no PCR.	Oligonucleotides covalently bound to quantum dots are required. The sensitivity is insufficient for clinical materials.	[51]
Using an on-chip nanowire sensor	100	It is possible to carry out a large number of different analyses of one specimen on a single chip. Telomerase binding and dissociation can be observed.	A transistor chip and the equipment to analyze it are required.	[53]
Bioluminescent method	5	Linear dependence of the signal on the amount of the telomerase-synthesized DNA. No radioactivity, no PAAG, no PCR. High-efficiency format.	The luciferase system for bioluminescence detection and a luminometer are required.	[55]
Electrochemiluminescence method	500	A high signal/noise ratio due to purification by modified magnetic bead extraction. High sensitivity.	It is difficult to synthesize a sample, requirements to the equipment.	[56]
Using FRET and total internal reflection fluorescence microscopy	1	Extremely high sensitivity – it is possible to detect the elongation of individual primers by telomerase. Possibility of combining this method with FRET-based methods for investigating the telomerase structure.	The method determines only the first 1.5 telomerase-synthesized repeats. It was tested only for*Tetrahymena thermophila*telomerase.	[57]
Using digoxigenin-labeled oligonucleotides complementary to telomeres (ELISA)	37,500	Quantitative determination; the method has been automated.	The sensitivity is insufficient for clinical materials.	[58]
Electrochemically, using avidin­–alkaline phosphatase conjugates	3,300	The absence of artifacts associated with PCR. High sensitivity. Rapid procedure.	A specially prepared electrode is needed. It is difficult to identify artifact signals if they emerge.	[47]
Using a fluorimetric optosensor	10^6^–10^7^	No radioactivity, no PAAG, no PCR.	Phosphotioate-modified oligonucleotides and an optic fiber system for fluorescence detection are required. The sensitivity is insufficient for analyzing clinical materials.	[59]
Using DNAzyme-labeled oligonucleotides	1,000	No radioactivity, no PAAG, no PCR. Simple procedure and short analysis time.	The specificity of the method is not very high; a background signal is present.	[61]
On the basis of the telomerase substrate that refolds in DNAzyme	10,000	No radioactivity, no PAAG, no PCR. A very simple procedure and short analysis time.	The sensitivity and specificity of the method are not very high; a background signal is present.	[60]
TRAP	10	Amplificates of telomerase-synthesized DNA can be observed. PAAG makes it possible to identify some PCR artifacts. High specificity.	Working with the radioactive label, necessity of using PAAG. PCR artifacts are possible.	[11]
TRAP from a single cell	1	High sensitivity. PAAG makes it possible to identify some PCR artifacts and qualitatively judge the processivity.	Working with the radioactive label, working with individual cells, necessity of using PAAG. PCR artifacts are possible.	[13]
TRAP with fluorescent primers	100	No radioactive labeling. PAAG makes it possible to identify some PCR artifacts and qualitatively judge the processivity.	Necessity of fluorescently labeled primers, necessity of using PAAG. PCR artifacts are possible.	[20]
TRAP with fluorescent staining of DNA in gel	10	No radioactive labeling. PAAG makes it possible to identify some PCR artifacts and qualitatively judge the processivity.	Necessity of using PAAG. PCR artifacts are possible. Most intercalating fluorescent dyes are mutagenic.	[16, 18]
TRAP with DNA staining with silver nitrite in gel	10	No radioactive labeling. PAAG makes it possible to identify some PCR artifacts and qualitatively judge the processivity.	Necessity of using PAAG. Possibility of PCR artifacts. High labor input.	[17]
TRAP with analyzing by scintillation proximity assay	10	No radioactivity, no PAAG. Highly efficient microplate format. Simple estimation of the amounts.	Necessity of using [^3^H]TTР and biotin-conjugated primers. Working with tritium. PCR artifacts are possible.	[27]
TRAP with detection by “hybridization protection”	10	No radioactivity, no PAAG. Highly efficient microplate format. Simple estimation of the amounts.	Acridine-labeled samples are required. PCR artifacts are possible.	[28]
TRAP with amplifluore primers	10-50	No radioactivity, no PAAG. Highly efficient microplate format. Quantitative assessment. Different labeling of amplificates by the telomerase-synthesized DNA and PCR control.	PCR artifacts are possible. Amplifluores are required.	[22]
TRAP combined with ELISA	10	No radioactivity, no PAAG. Highly efficient microplate format.	PCR and ELISA artifacts are possible.	[29]
TRAP with primers with fluorescence resonance energy transfer(FRET)	10	No radioactivity, no PAAG. Highly efficient microplate format.	Unequal consideration of the first and subsequent repeats synthesized by telomerase when carrying out the quantitative assessment. PCR artifacts are possible. Special fluorophores are required.	[26]
TRAP on microchips	10	No radioactivity, no PAAG. Small reaction volumes. Highly efficient microplate format. Simple estimation of the amounts.	Chips and an instrument to read them are necessary.	[35]
TRAP with real-time PCR	50	No radioactivity, no PAAG. Highly efficient microplate format. Simple estimation of the amounts. Different labeling of amplificates of the telomerase-synthesized DNA and PCR control with amplifluores is possible.	An amplifier for real-time PCR is required. The identification of PCR artifacts is complicated.	[32, 33]
*In situ*TRAP	1	The method provides information on the telomerase activity in individual cells and some information on endocellular localization of the activity.	A complicated procedure. Low efficiency. A fluorescence microscope is required. The method has been optimized only for analyzing clinical samples and cell lines.	[36]
TRAP with transcriptional amplification	10	Isothermal amplification, no PCR artifacts, PAAG-free detection, highly efficient format.	Acridine-labeled samples are required. Artifacts of transcriptional amplification are possible. Higher requirements of purity (absence of RNases) because of the transcriptional amplification.	[42]

* The minimum number of cells of telomerase-positive cell lines for which activity can be detected.

** In PAAG-free methods (with the exception of determination of telomerase activity using FRET and total internal reflection fluorescence microscopy), polymerase activity (but not processivity) is assessed.

Similarly, a DNAzyme that is covalently linked with the antisense oligonucleotide can be used for the telomerase-synthesized DNA [61] ( *Fig. 20B* ). It is virtually similar to ELISA in which an enzyme has already been covalently linked to the probe. The sensitivity of this modification is approximately 1,000 HeLa cells upon a lower background. The drawbacks of the method include low sensitivity and specificity, as well as the presence of a background signal.

##  CONCLUSIONS 

At the time of publication of this article, there were a number of methods for the determination of telomerase activity in various specimens: extracts of cells, tissues, and mixed cell populations. All the aforementioned methods can be divided into two groups: those with direct detection of telomerase-synthesized DNA and those with various amplification schemes; each method has its advantages and drawbacks. A comparison of all these methods is provided in *Table 2* .

 After the consideration of all the methods, a number of new systems can be singled out which are comparable in terms of sensitivity with the TRAP method, the most commonly used method at the moment. They do not have artifacts associated with amplification; the analysis is carried out at a high rate with high sensitivity. Meanwhile, they have a number of drawbacks, including high requirements to the specific supplies and equipment. Therefore, the selection of a technique in each particular case can be determined by the availability of the equipment and reagents. In a number of methods, it is difficult to distinguish between the specific and nonspecific signals. Most methods assess only telomerase activity, but not its processivity. They frequently provide no information on the length distribution of the telomerase-synthesized DNA. Some methods are highly specialized; e.g., the method in which telomerase-synthesized DNA is determined using dioxigenin-labeled oligonucleotides that are complementary to telomeres (ELISA) without PCR is far from being the most sensitive method; however, it has been completely automated and optimized for the search for telomerase inhibitors. The determination of telomerase activity by FRET and total internal reflection fluorescence microscopy makes it possible to detect the elongation of individual primers by telomerase, which can be combined with FRET-based methods for investigating the telomerase structure; however, it allows detection of only the first 1.5 telomerase-synthesized repeats and is intended for research purposes. 

## References

[1] Hayflick L., Moorhead P.S. (1961). Exp. Cell Res..

[2] Olovnikov A.M. (1973). J. Theor. Biol..

[3] Blackburn E.H. (2001). Cell..

[4] Pandita T.K., Hunt C.R., Sharma G.G., Yang Q. (2007). Cell Mol. Life Sci..

[5] Greider C.W., Blackburn E.H. (1985). Cell..

[6] Blackburn E.H. (1991). Nature..

[7] Maida Y., Yasukawa M., Furuuchi M., Lassmann T., Possemato R., Okamoto N., Kasim V., Hayashizaki Y., Hahn W.C., Masutomi K. (2009). Nature..

[8] Pasrija T., Srinivasan R., Behera D., Majumdar S. (2007). Eur. J. Cancer..

[9] Kedde M., le Sage C., Duursma A., Zlotorynski E., van Leeuwen B., Nijkamp W., Beijersbergen R., Agami R. (2006). J. Biol. Chem..

[10] Skvortsov D.A., Rubtsova M.P., Zvereva M.E., Kisseljov F.L., Dontsova O.A. (2009). Acta Naturae,.

[11] Kim N.W., Piatyszek M.A., Prowse K.R., Harley C.B., West M.D., Ho P.L., Coviello G.M., Wright W.E., Weinrich S.L., Shay J.W. (1994). Science..

[12] Saldanha S.N., Andrews L.G., Tollefsbol T.O. (2003). Anal. Biochem..

[13] Wright W.E., Shay J.W., Piatyszek M.A. (1995). Nucl. Acids Res..

[14] Skvortsov D.A., Gasparyan N.M., Rubtsova M.P., Zvereva M.E., Fedorova M.D., Pavlova L.S., Bogdanov A.A., Dontsova O.A., Kisseljov F.L. (2006). Doklady Akademii Nauk,.

[15] Gomez D., Mergny J.L., Riou J.F. (2002). Cancer Res..

[16] Gan Y., Lu J., Johnson A., Wientjes M.G., Schuller D.E., Au J.L. (2001). Pharm. Res..

[17] Dalla Torre C.A., Maciel R.M., Pinheiro N.A., Andrade J.A., De Toledo S.R., Villa L.L., Cerutti J.M. (2002). Braz. J. Med. Biol. Res..

[18] Holt S.E., Norton J.C., Wright W.E., Shay J.W. (1996). Methods Cell. Sci..

[19] Skvortsov D.A., Zvereva M.E., Pavlova L.S., Petrenko A.A., Kisseljov F.L., Dontsova O.A. (2010). Vestn. MSU,.

[20] Aldous W.K., Grabill N.R. (1997). Diagn. Mol. Pathol..

[21] Gollahon L.S., Holt S.E. (2000). Cancer Lett..

[22] Uehara H., Nardone G., Nazarenko I., Hohman R.J. (1999). Biotechniques..

[23] Szatmari I., Tokes S., Dunn C.B., Bardos T.J., Aradi J. (2000). Anal. Biochem..

[24] Szatmari I., Aradi J. (2001). Nucl. Acids Res..

[25] Bazin H., Preaudat M., Trinquet E., Mathis G. (2001). Spectrochim. Acta A Mol. Biomol. Spectrosc..

[26] Gabourdes M., Bourgine V., Mathis G., Bazin H., Alpha-Bazin B. (2004). Anal. Biochem..

[27] Savoysky E., Akamatsu K., Tsuchiya M., Yamazaki T. (1996). Nucl. Acids Res..

[28] Hirose M., Abe-Hashimoto J., Ogura K., Tahara H., Ide T., Yoshimura T. (1997). J. Cancer Res. Clin. Oncol..

[29] Mayfield M.P., Shah T., Flannigan G.M., Hamilton Stewart P.A., Bibby M.C. (1998). Int. J. Mol. Med..

[30] Hoos A., Hepp H.H., Kaul S., Ahlert T., Bastert G., Wallwiener D. (1998). Int. J. Cancer..

[31] Chen L., Huang J., Meng F., Zhou N. (2010). Anal. Sci..

[32] Hou M., Xu D., Bjorkholm M., Gruber A. (2001). Clin. Chem..

[33] Elmore L.W., Forsythe H.L., Ferreira-Gonzalez A., Garrett C.T., Clark G.M., Holt S.E. (2002). Diagn. Mol. Pathol..

[34] Kong D., Jin Y., Yin Y., Mi H., Shen H. (2007). Anal. Bioanal. Chem..

[35] Heller-Uszynska K., Kilian A. (2004). Biochem. Biophys. Res. Commun..

[36] Ohyashiki K., Ohyashiki J.H., Nishimaki J., Toyama K., Ebihara Y., Kato H., Wright W.E., Shay J.W. (1997). Cancer Res..

[37] Yahata N., Ohyashiki K., Ohyashiki J.H., Iwama H., Hayashi S., Ando K., Hirano T., Tsuchida T., Kato H., Shay J.W. (1998). J. Natl. Cancer Inst..

[38] Ohyashiki K., Yahata N., Ohyashiki J.H., Iwama H., Hayashi S., Ando K., Aizawa T., Ito T., Miki M., Ebihara Y. (1998). Cancer..

[39] Dejmek A., Yahata N., Ohyashiki K., Kakihana M., Hirano T., Kawate N., Kato H., Ebihara Y. (2000). Cancer..

[40] Dejmek A., Yahata N., Ohyashiki K., Ebihara Y., Kakihana M., Hirano T., Kawate N., Kato H. (2001). Diagn. Cytopathol..

[41] Youssef N., Paradis V., Ferlicot S., Bedossa P. (2001). J. Pathol..

[42] Hirose M., Abe-Hashimoto J., Tahara H., Ide T., Yoshimura T. (1998). Clin. Chem..

[43] Blackburn E.H., Greider C.W., Henderson E., Lee M.S., Shampay J., Shippen-Lentz D. (1989). Genome..

[44] Maesawa C., Inaba T., Sato H., Iijima S., Ishida K., Terashima M., Sato R., Suzuki M., Yashima A., Ogasawara S. (2003). Nucl. Acids Res..

[45] Rad’ko S.P., Voronina S.A., Gromov A.V., Gnedenko O.V., Bodoev N.V., Ivanov A.S., Yarygin K.N. (2009). Bull. Exp. Biol. Med..

[46] Grimm J., Perez J.M., Josephson L., Weissleder R. (2004). Cancer Res..

[47] Pavlov V., Willner I., Dishon A., Kotler M. (2004). Biosens. Bioelectron..

[48] Sato S., Kondo H., Nojima T., Takenaka S. (2005). Anal. Chem..

[49] Li Y., Liu B., Li X., Wei Q. (2010). Biosens. Bioelectron..

[50] Schmidt P.M., Matthes E., Scheller F.W., Bienert M., Lehmann C., Ehrlich A., Bier F.F. (2002). Biol. Chem..

[51] Patolsky F., Gill R., Weizmann Y., Mokari T., Banin U., Willner I. (2003). J. Am. Chem. Soc..

[52] Sharon E., Freeman R., Willner I. (2010). Anal. Chem..

[53] Zheng G., Patolsky F., Cui Y., Wang W.U., Lieber C.M. (2005). Nat. Biotechnol..

[54] Sharon E., Freeman R., Riskin M., Gil N., Tzfati Y., Willner I. (2010). Anal. Chem..

[55] Xu S.Q., He M., Yu H.P., Wang X.Y., Tan X.L., Lu B., Sun X., Zhou Y.K., Yao Q.F., Xu Y.J. (2002). Clin.Chem..

[56] Zhou X., Xing D., Zhu D., Jia L. (2009). Anal. Chem..

[57] Wu J.Y., Stone M.D., Zhuang X. Nucl. Acids Res. V..

[58] Kha H., Zhou W., Chen K., Karan-Tamir B., San Miguel T., Zeni L., Kearns K., Mladenovic A., Rasnow B., Robinson M. (2004). Anal. Biochem..

[59] Schmidt P.M., Lehmann C., Matthes E., Bier F.F. (2002). Biosens. Bioelectron..

[60] Xiao Y., Pavlov V., Niazov T., Dishon A., Kotler M., Willner I. (2004). J. Am. Chem. Soc..

[61] Pavlov V., Xiao Y., Gill R., Dishon A., Kotler M., Willner I. (2004). Anal. Chem..

